# High-Quality Nutritional and Medical Care in Celiac Disease Follow-Up

**DOI:** 10.3390/nu17223530

**Published:** 2025-11-11

**Authors:** Anthony Kerbage, Claire Jansson-Knodell, Kendra Weekley, David Gardinier, Alberto Rubio-Tapia

**Affiliations:** 1 Department of Internal Medicine, Cleveland Clinic, Cleveland, OH 44195, USA; kerbaga@ccf.org; 2 Celiac Disease Program, Department of Gastroenterology, Hepatology and Nutrition, Cleveland Clinic, Cleveland, OH 44195, USA

**Keywords:** celiac disease, gluten-free diet, follow-up care, multidisciplinary care, nutritional management, micronutrient deficiencies, metabolic syndrome, mucosal healing, quality of life, preventive health

## Abstract

**Background:** Celiac disease (CeD) is a chronic, immune-mediated enteropathy triggered by gluten ingestion in genetically predisposed individuals. While a lifelong gluten-free diet (GFD) remains the cornerstone of treatment, inadequate follow-up can lead to persistent symptoms, nutritional deficiencies, and long-term complications. **Aim:** This narrative review summarizes best practices in celiac disease follow-up, with emphasis on multidisciplinary, nutritional, clinical, and preventive care strategies to optimize long-term outcomes. **Main Findings:** High-quality follow-up requires coordinated care involving gastroenterologists, dietitians, primary care providers, and other specialists. Nutritional challenges of the GFD include high cost, limited fortification, suboptimal nutrient content, and increased risk of obesity and metabolic dysfunction. Patients also face psychosocial and behavioral burdens such as anxiety, social isolation, and disordered eating. Evidence-based strategies for follow-up include structured clinical and serologic monitoring, laboratory assessments, bone health evaluation, cancer risk reduction, and preventive care. Novel tools such as gluten immunogenic peptide testing, digital health platforms, and artificial intelligence are emerging as adjuncts to clinical management. **Implications:** Structured, patient-centered follow-up that integrates medical, nutritional, and psychosocial dimensions is essential to achieving mucosal healing, maintaining long-term health, and improving quality of life in individuals with CeD.

## 1. Introduction

Celiac disease (CeD) is a chronic immune-mediated enteropathy precipitated by exposure to dietary gluten in genetically predisposed individuals and affects approximately 1% of the global population, though many remain undiagnosed [1]. The cornerstone of CeD treatment is a lifelong gluten-free diet (GFD), which typically leads to symptom resolution, serologic normalization, and mucosal healing [2]. However, adherence can be challenging, and inadequate follow-up may result in persistent symptoms, nutritional deficiencies, and long-term complications. Therefore, structured follow-up care is essential to ensure dietary compliance, monitor recovery, and prevent adverse outcomes. The primary goals of follow-up include symptom control, celiac-associated antibody normalization, mucosal healing, prevention of complications, and maintenance of nutritional and general health. Although several reviews have addressed CeD management, the landscape has recently evolved with the 2023 American College of Gastroenterology (ACG) guidelines [2] and, for the first time, dedicated international guidelines on CeD follow-up [3], providing updated recommendations for diagnosis and management while also establishing consensus on structured follow-up, an area previously lacking formal guidance. Together, these developments highlight the need for an updated summary and provide the basis for this review.

We aim to provide a comprehensive overview of best practices in CeD follow-up care, with a focus on multidisciplinary management, nutritional monitoring, clinical follow-up, and emerging strategies to optimize long-term health outcomes. We aim to provide a practical and evidence-based overview of high-quality longitudinal CeD patient care.

## 2. Methods

This review was conducted as a narrative overview of the literature on CeD follow-up care in adults. Relevant articles were identified through searches of PubMed using combinations of keywords such as *celiac disease*, *follow-up*, *nutritional management*, *multidisciplinary care*, and *long-term outcomes*. The search included publications from 2000 through 2025, with emphasis on recent clinical guidelines, systematic reviews, and original research. Additional references were identified from bibliographies of key articles and guidelines, as well as the authors’ expertise in clinical practice and research. Both United States (US) and international literature were considered, although the primary focus is on US-based protocols and practice patterns. Inclusion criteria were relevant articles that encompassed the following article types: original research articles, clinical guidelines, systematic reviews, and narrative reviews focused on adult CeD follow-up, nutritional monitoring, multidisciplinary management, or preventive care. Exclusion criteria included articles that were case reports, pediatric-only studies, conference abstracts without full data, and non-English-language papers. Because this is a narrative review, formal systematic screening or quality grading of evidence was not performed. In addition to the published literature, our practical recommendations and areas of emphasis were informed by our clinical experience delivering multidisciplinary care at a high-volume CeD center in the US. These experiential insights were used to contextualize and operationalize the evidence (e.g., clinic workflows, referral triggers, and monitoring intervals) but did not substitute for published data when available.

## 3. Multidisciplinary Approach to Celiac Disease Follow-Up

Effective management of CeD requires a collaborative, multidisciplinary approach (Table 1). The gastroenterologist plays a central role in confirming the diagnosis, initiating the GFD, and monitoring disease activity through clinical assessments, serologic testing, and, when indicated, repeat endoscopy. Regular evaluation is crucial to identify ongoing gluten exposure, refractory disease, micronutrient deficiencies, or associated conditions such as dermatitis herpetiformis, microscopic colitis, or pancreatic insufficiency [4].

Registered dietitians are vital team members, providing personalized dietary counseling to ensure strict GFD adherence, educate patients on food labeling, cross-contamination, and the importance of nutritional adequacy. They are also instrumental in identifying and correcting dietary gaps. Dietitians are advised to implement SMART (Specific, Measurable, Achievable, Relevant, Time-bound) goals that take into account patient literacy, cultural norms, and socioeconomic barriers [5]. Beyond ensuring gluten avoidance, dietitians play a pivotal role in supporting patients with symptom management, meal planning, and behavioral counseling. This includes addressing comorbid food intolerances (e.g., lactose, FODMAPs), guiding fiber intake to manage constipation, and counseling on balanced, sustainable nutrition [5]. Dietitian techniques such as motivational interviewing and cognitive behavioral therapy have been shown to enhance dietary adherence and coping skills [6]. Additionally, strategies like mindful and intuitive eating are increasingly being used by dietitians to help patients foster a healthy relationship with food while navigating the complexities of a lifelong GFD [7]. Patients should be referred to a dietitian at the time of diagnosis and during follow-up of patients in general, but particularly those with persistently elevated or detectable celiac serology, persistent symptoms, difficulty with GFD adherence, or poor GFD nutritional quality.

Additional specialists may be involved depending on the clinical context. Endocrinologists may assist with bone health management, especially in patients with osteopenia or osteoporosis. Psychologists or mental health professionals may address the psychological burden of CeD and the dietary restrictions it imposes. Pharmacists play a key role in reviewing medications for potential gluten content, while primary care providers contribute to the coordination of preventive care and general health maintenance. Other relevant specialists may include dermatologists for those with dermatitis herpetiformis, neurologists for those with gluten ataxia, hematologists for those with iron deficiency requiring intravenous iron replacement, and obstetricians for those pregnant CeD patients.

**Figure 1:**
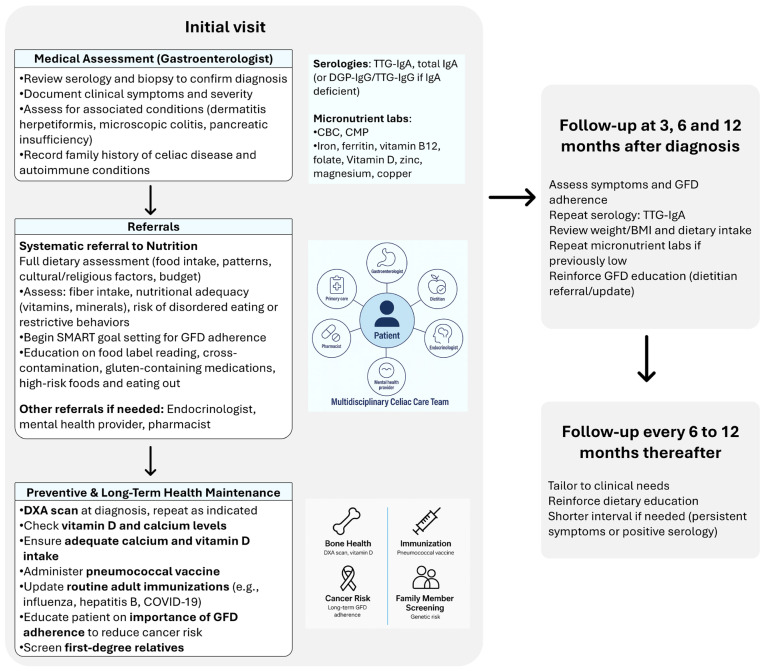
Structured Follow-Up and Monitoring Timeline for Patients with Celiac Disease.

Dedicated CeD centers or specialized clinics have emerged to provide comprehensive care (Figure 1). Studies have shown that patients managed through such programs are more likely to achieve mucosal healing and report higher quality of life and dietary adherence. The integration of multidisciplinary expertise into a structured follow-up model can improve long-term outcomes and patient satisfaction [8].

## 4. Nutritional Management and Challenges in Celiac Disease

The GFD remains the cornerstone of therapy for CeD [2], yet it is far from a simple dietary adjustment (Table 2). When not carefully planned, it is associated with adverse nutritional, metabolic, and psychosocial consequences.

First, cost and accessibility remain major barriers to adhering to a GFD [9,10]. Across studies from multiple regions, gluten-free products are consistently more expensive than their gluten-containing counterparts, typically by 150–300% on average, although this varies substantially depending on the type of product and market. In the US, market basket analyses show gluten-free staples such as bread and pasta costing more than twice as much as regular versions [10]. Comparable studies in the United Kingdom reported gluten-free foods to be ~159% more expensive on average [11,12]. Similar patterns were observed across southern Europe (Greece, Cyprus, Turkey), where gluten-free alternatives were reported to be 22–476% more expensive [13,14,15]. In Latin America, gluten-free basic food baskets were found to be threefold more costly and up to 42% less available than regular baskets, especially in lower-income areas [9]. Importantly, the methodology of these surveys varies: most used a “basic food basket” approach or systematic online price collection, focusing on the lowest-cost equivalent gluten-free items within key food categories such as bread, cereals, and pasta. As a result, direct cross-country comparisons should be interpreted cautiously.

Moreover, enrichment and reimbursement policies differ widely. For example, the United Kingdom previously offered partial reimbursement for gluten-free staple foods through prescription schemes, although these benefits have been scaled back in recent years. Argentina, Italy, Greece, Norway, and Sweden provide a monthly stipend to offset the costs of gluten-free foods. Amount of payment, form of payment, and products covered vary. A medical deduction on taxes is available in the US and Canada. Many countries lack such programs, placing a disproportionate economic burden on affected individuals. Collectively, these findings highlight persistent global inequities in the cost, availability, and nutritional quality of gluten-free foods.

These high costs are not just a matter of inconvenience; they directly affect dietary adherence and quality of life. In one study, 33% of patients cited cost as a primary reason for nonadherence to the GFD [16], and in another study the overall treatment burden of CeD was rated as higher than that of diabetes or congestive heart failure [17]. Furthermore, availability is inconsistent: while large coastal cities offer a broader selection, options are much more limited in less urban areas and certain regions [18]. Although the number of gluten-free products in traditional grocery stores has increased, the variety of brands has declined, with national mass-market producers replacing smaller, regional brands [19]. These ongoing challenges can contribute to food insecurity, reduced dietary variety, and significant disparities in access to dietary treatment for individuals with CeD [19,20].

Second, the nutritional quality of many processed gluten-free foods is suboptimal (Table 2). Unlike their gluten-containing counterparts, gluten-free products are often not fortified with essential micronutrients such as folic acid, thiamine, and iron [20,21]. This lack of fortification contributes to persistent deficiencies despite dietary adherence and underscores the importance of comprehensive nutritional assessments even in asymptomatic patients. Moreover, these products often contain excessive sugars, saturated fats, and sodium, while being low in fiber and other nutrients [3]. As a result, many individuals with CeD experience undesired weight gain and metabolic complications (Table 2). A meta-analysis estimates that 15–31% of patients are overweight and 6.8–13% are obese at the time of diagnosis [22]. After adopting a GFD, an additional ~9% of patients transition from underweight or normal BMI to overweight or obese [23]. This weight gain is likely driven by increased absorption post-healing, the poor nutritional profile of many gluten-free products, and an overall rise in caloric intake when the patient feels better. Long-term risks include metabolic dysfunction-associated steatotic liver disease (MASLD), insulin resistance, and cardiovascular disease [24]. In a prospective Italian cohort of 185 adults with CeD, the prevalence of metabolic syndrome increased from 3.2% at diagnosis to 14.6% after ≥1 year on GFD, while MASLD rose from 1.7% to 11.1% [25]. Similarly, a multicenter cohort of 221 CeD patients found that the prevalence of MASLD doubled after two years of GFD, from 14.5% to 32.6%, with affected patients demonstrating higher BMI, waist circumference, and insulin resistance compared with baseline [26]. A 2022 meta-analysis including 11 studies (*n* = 2578) reported pooled prevalences of 18.2% (95% CI, 8.3–30.8%) for MASLD and 4.3% (95% CI, 2.4–6.7%) for metabolic syndrome in treatment-naïve patients, rising to 28.2% (95% CI, 20.7–36.4%) and 21.3% (95% CI, 11.7–32.9%) after GFD [27]. These trends suggest that celiac patients are not exempt from the obesity epidemic and may even be particularly vulnerable due to the nutritional composition of gluten-free foods. Routine counseling on physical activity, calorie balance, and the quality of macronutrient intake should therefore be integrated into long-term nutrition care plans.

In addition to these dietary challenges, nutritional screening is critical in CeD but remains underdeveloped. Standard malnutrition screening tools such as the Nutritional Risk Screening 2002 (NRS-2002), the Malnutrition Universal Screening Tool in the community (MUST), and Mini Nutritional Assessment in the care of older adults—Short Form (MNA-SF) are widely used in clinical practice [28], but none are validated specifically for CeD. These tools overlook factors unique to celiac patients, like gluten-related inflammation and GFD adherence, that directly affect nutritional status. Emerging tools, such as the Celiac Disease Adherence Test (CDAT) [29] and Biagi score [30], provide insight into adherence, but expert dietitian review remains the gold standard for nutritional and adherence assessment. The lack of disease-specific screening tools represents a critical gap in optimizing follow-up care.

Third, micronutrient deficiencies are both common at diagnosis and persistent during follow-up. These include iron, folate, vitamins D, and B12, zinc, and copper [31]. As mentioned above, gluten-free products often lack fortification (Table 2); additionally, mucosal healing can take years to occur, and it may be incomplete. For this reason, laboratory monitoring should be performed regularly. Importantly, laboratory results should be interpreted alongside inflammatory markers such as *C*-reactive protein (CRP), which can be elevated in patients with CeD who also have infections or other inflammatory conditions. Elevated CRP may distort the interpretation of certain nutrient levels, such as by raising ferritin levels and masking iron deficiency. It can also lead to the false appearance of deficiencies in nutrients like selenium, vitamins A, D, and C, and zinc [5]. This can result in inappropriate supplementation, potentially causing nutrient excess or even secondary deficiencies, such as copper deficiency following excessive zinc intake.

Supplementation should be individualized based on intake and lab results. It is recommended to recheck labs 3 to 6 months after supplementation is initiated and then monitor annually or biennially depending on stability. A standard lab panel might include iron studies, ferritin, vitamin D, folate, B12, zinc, and copper [5].

Fourth, the GFD can impose significant psychosocial burdens [32]. Patients may experience food-related anxiety, disordered eating, and social isolation (Table 2). Past studies have highlighted increased prevalence of eating disorders in patients with CeD, including anorexia nervosa and avoidant/restrictive food intake disorder (ARFID) that may be due to fear of contamination [33,34,35,36]. Beyond food-related fears, the social and emotional toll of a GFD may be substantial, especially in the context of dating. In a 2022 study, nearly 70% of adults with CeD reported that the disease had a major or moderate impact on their dating life [37]. Many expressed hesitancy about going on dates (48%), discomfort discussing dietary precautions in front of partners (39%), and even avoided kissing due to fears of gluten transfer (39%), despite minimal evidence supporting a clinically meaningful risk (33%). Notably, 28% admitted to engaging in riskier eating behaviors on dates, and 7.5% intentionally consumed gluten, highlighting the intense social pressures involved [37]. These challenges may be associated with higher social anxiety, lower quality of life, and less adaptive eating attitudes. Education that avoids fear-based messaging and promotes inclusive, flexible strategies is critical, especially for young adults navigating dating and intimacy while managing CeD.

Given the complex interplay of these factors, structured monitoring and multidisciplinary care are essential (Figure 1). Routine assessments should include dietitian-led food recalls, body composition analysis, and individualized dietary planning. Barriers such as food cost, access, and adherence challenges should be addressed through counseling and referrals to social support services when necessary. Dietitians can help patients optimize fiber intake, manage symptoms like constipation, and ensure nutritional adequacy. In partnership with the dietitians, physicians should screen for bone loss in those with suboptimal calcium and vitamin D levels, and for MASLD and metabolic syndrome in those who are overweight or obese.

## 5. Medical Management

Next, we will turn our attention to clinical and serologic monitoring for the follow-up of CeD (Figure 1). This will be followed by health maintenance issues specific to the CeD population.

### 5.1. Clinical and Serologic Monitoring

Most guidelines support a structured follow-up schedule. During the first year after diagnosis, patients may require visits at approximately 3, 6, and 12 months to assess symptom response, review serologic markers, evaluate nutritional status, and reinforce dietary education [2]. After the first year, follow-up visits are generally recommended every 6 to 12 months, tailored to individual clinical needs. Patients with persistent symptoms, ongoing serologic elevation, or suspected nonadherence may benefit from shorter follow-up intervals (e.g., every 6 months) until disease control is achieved [2,3,38] (Figure 1).

For dietician follow-up, the 2023 Academy of Nutrition and Dietetics guidelines do not recommend fixed intervals but emphasize individualized assessment based on patient needs and disease activity [20].

Tissue transglutaminase IgA (TTG-IgA) serology should be used routinely to detect gluten exposure, but not as a marker of mucosal healing due to poor sensitivity [2]. Persistently elevated TTG-IgA levels suggest ongoing gluten exposure, while normalized values cannot confirm adherence.

Duodenal biopsies are not recommended routinely but are indicated in patients with persistent symptoms, positive serology despite diet adherence, or suspicion of refractory CeD [2,3]. When performed, biopsies should include four samples from the second part of the duodenum and one to two from the bulb. The latest CeD guidelines recommend treatment of CeD to a goal of intestinal healing [2]. With that goal in mind, patients and physicians can use shared decision making to consider a follow-up biopsy in asymptomatic patients with normal serology after 2 years on a GFD to assess small intestinal health and document intestinal healing. When this goal is achieved, repeat procedures or biopsies are not needed unless their clinical picture were to change.

As discussed earlier, micronutrient testing (e.g., iron, B12, folate, vitamin D, zinc) should be personalized based on baseline deficiencies, with routine testing for iron status and complete blood count particularly in women of reproductive age. Monitoring for metabolic complications (e.g., MASLD, metabolic syndrome) is also warranted during follow-up.

Urinary and fecal gluten immunogenic peptide (GIP) testing is an increasingly recognized, noninvasive tool for detecting gluten exposure, particularly in patients with nonresponsive CeD. These biomarkers can identify low-level gluten ingestion not evident by symptoms or serology, offering a direct and objective tool for dietary assessment [2,3]. It is particularly valuable because many patients with persistent mucosal damage are asymptomatic and seronegative, yet GIP-positive [3,39]. However, interpretation of GIP testing requires caution: detection sensitivity varies by dose of gluten, rate of digestion, and matrix (higher for stool than urine), and results are affected by timing, short detection windows, and interindividual variability. False negatives may occur with very low gluten intake or suboptimal timing of test performance, and false positives may occur from assay variability or contamination [2,3,40,41]. A positive GIP should prompt review of dietary practices with a dietitian, reassessment of serology and nutritional status. Repeated negative GIP results, particularly in asymptomatic and seronegative patients, strongly predict mucosal healing and may reduce the need for biopsy [2,3,39,41]. Selective use of GIP testing is an additional tool, ideally an adjunct to—not a replacement for—traditional adherence assessments [2,3]. They provide an objective correlate for evaluation of voluntary or involuntary gluten exposure to complement subjective dietary reviews.

Standardized questionnaires such the Celiac Dietary Adherence Test (CDAT) and the Biagi questionnaire can be used to better understand GFD adherence and when dietitian expertise is unavailable [5,42]. However, expert dietitian evaluation remains the gold standard for both adherence and nutritional quality assessment. The physician is primarily responsible for evaluating for symptom improvement with the start of treatment, ensuring referral for the opportunity to meet with a dietitian with GFD expertise, performing laboratory monitoring, and seeing regularly for clinical follow-up.

### 5.2. Preventive and Long-Term Health Maintenance

CeD is associated with long-term health risks that require vigilant preventive care (Figure 1). Osteoporosis is a notable concern due to impaired calcium and vitamin D absorption. Baseline bone mineral density testing with dual-energy X-ray absorptiometry (DXA) is recommended at diagnosis in adults, especially postmenopausal women and men over 50, and repeat testing should be individualized but is commonly done 2 years after an abnormal scan to assess for improvement on a GFD or a trajectory of worsening despite appropriate dietary management. While vitamin D deficiency is common, its correlation with bone density is inconsistent, so decisions regarding supplementation should be based on serum levels and clinical context [3]. Referral to endocrinology is recommended for those with bone health readings outside of the expected range for age.

Vaccination against pneumococcal infection is recommended due to functional hyposplenism observed in some patients with CeD [43]. In the US, the Centers for Disease Control and Prevention (CDC) considers patients fully vaccinated after receiving either a single dose of PCV20 or a sequential regimen of PCV15 followed by PPSV23, depending on age and comorbidities. Local immunization schedules and revaccination intervals vary internationally; therefore, clinicians should follow region-specific vaccination guidelines and formulations. Other routine immunizations should be updated per general population guidelines [2,43].

Although the absolute cancer risk in CeD remains low, studies consistently show an increased relative risk of certain malignancies, most notably enteropathy-associated T-cell lymphoma (EATL), other T-cell non-Hodgkin lymphomas, and small-bowel adenocarcinoma, particularly among patients with persistent villous atrophy compared with those achieving mucosal healing [44,45,46,47,48,49]. This increased risk is most evident in individuals diagnosed after age 40 and is most pronounced within the first year following diagnosis [50]. These associations are derived from observational cohorts and are subject to confounding by indication, diagnostic delay, surveillance bias, and disease severity. Adherence to a strict GFD reduces this risk, particularly when it results in mucosal healing, while persistent villous atrophy from ongoing gluten exposure or refractory disease increases the risk of lymphoproliferative malignancy [44,48,50,51,52]. The extent to which a GFD reduces this risk in asymptomatic or well-healed individuals remains uncertain. Cases of EATL and small-bowel carcinoma have been reported even in patients adhering to a GFD [2,47,53]. These are, however, uncommon and may reflect delayed diagnosis or prolonged pre-treatment exposure.

Finally, first-degree relatives of patients with CeD carry an elevated genetic risk for the condition. Systematic screening using serologic testing is advised (Figure 1), especially in symptomatic individuals or those with other autoimmune conditions [2]. This screening is particularly relevant for those adult patients with children who are growing. Intestinal and extra-intestinal symptoms (especially growth concerns) should prompt testing.

## 6. Future Directions

### 6.1. Nondietary and Adjunctive Pharmacologic Therapies

While a strict GFD remains the cornerstone of CeD management, several nondietary and adjunctive therapies are being explored to address persistent challenges with adherence, inadvertent gluten exposure, and incomplete mucosal healing [54,55,56,57]. Among these, gluten-degrading enzymes (such as AN-PEP and latiglutenase) aim to break down immunogenic peptides before they reach the small intestine, potentially reducing harm from trace gluten exposure [58,59].

Tight-junction modulators like larazotide seek to reduce intestinal permeability and subsequent immune activation [60] showing modest symptom improvement in early studies but variable results in larger trials [61,62,63]. Other investigational approaches include transglutaminase-2 inhibitors [64,65], HLA-DQ blockers [66,67], and immune-tolerizing medications and vaccines [68,69,70], which target key steps in the celiac immune cascade and could eventually modify disease biology. Microbiome-directed therapies, such as probiotics or prebiotics, are also under investigation, aiming to rebalance intestinal microbial composition and immune signaling [71,72,73].

### 6.2. Digital Health, Remote Monitoring, and AI

Digital health and artificial intelligence (AI) are also transforming the landscape of follow-up care. Telehealth visits, mobile applications, and barcode-scanning tools can support patient education, symptom tracking, and label literacy, helping to overcome barriers related to cost, access, and dietary complexity [74,75,76,77,78,79]. In addition, electronic health record (EHR)-based alerts and automated care pathways can streamline multidisciplinary coordination, ensuring timely dietitian review, bone health screening, and preventive care, although direct evidence for their impact on CeD outcomes is limited. AI-driven chatbots also represent an emerging tool to support patient engagement and education. They can assist with answering common questions about CeD, explaining dietary principles, generating gluten-free meal ideas, and reinforcing follow-up recommendations, serving as accessible adjuncts that extend patient support beyond clinic visits [80]. However, these innovations also carry important limitations. The quality of evidence remains preliminary as most studies are proof-of-concept or small randomized controlled trials with short-term outcomes, and long-term benefits on adherence, mucosal healing, or cost-effectiveness are not yet established. Many digital applications still lack formal validation or professional oversight, and their implementation raises concerns related to data privacy, algorithmic transparency, and ethical use of patient information. Interoperability challenges and unequal access driven by digital literacy and socioeconomic disparities may further limit their real-world impact. In addition, current AI chatbots often provide incomplete or inaccurate information, underscoring the need for clinician supervision and rigorous quality control.

Overall, both pharmacologic and digital innovations hold promise for making CeD management more personalized, accessible, and proactive. However, until further validated by large-scale, patient-centered studies demonstrating sustained improvements in adherence, mucosal healing, and quality of life, these tools should be viewed as adjuncts, not substitutes, to evidence-based multidisciplinary follow-up and a well-supported GFD.

## 7. Limitations

This review has several limitations. First, it is a narrative review and does not employ a systematic methodology such as PRISMA guidelines, nor does it include a formal grading of evidence quality. As such, the selection of studies may be subject to selection bias, as inclusion was influenced by availability, relevance, and author judgment, and the synthesis reflects the interpretive nature of a narrative review based on expert perspective. It should be noted that in the recent CeD follow-up guidelines all recommendations are supported by low or very low quality of evidence [3] and low quality of evidence in the American College of Gastroenterology guidelines per GRADE system [2]. Second, while we aimed to provide a comprehensive and clinically oriented overview of CeD follow-up, the review is not exhaustive, and some relevant studies may not have been included. Third, although both US and international literature were considered, there is an emphasis on US practice patterns and guidelines given the authors’ clinical experience in that practice setting, which may limit generalizability to other healthcare settings. Despite these limitations, this review synthesizes current knowledge and highlights practical strategies the authors use in our own clinical practice at a high-volume celiac disease program in the United States to optimize long-term care in individuals with CeD.

## 8. Conclusions

High-quality follow-up in CeD requires more than gluten elimination; it depends on a structured, multidisciplinary approach that integrates gastroenterologists, dietitians, and primary care providers to ensure adherence, timely monitoring, and prevention of complications. Attention to nutritional and psychosocial challenges is critical, as patients often face persistent micronutrient deficiencies, weight gain, and anxiety related to the gluten-free diet, underscoring the need for ongoing counseling, laboratory assessment, and mental health support. Emerging tools such as gluten immunogenic peptide testing, digital health platforms, and behavioral interventions hold promise as adjuncts to traditional care, providing opportunities to personalize management and improve long-term outcomes and quality of life. Finally, pharmacotherapy is an active area of investigation but no FDA-approved medication is currently available.

## Figures and Tables

**Table 1:** Roles of Professionals in Celiac Disease Follow-Up.

Professional	Key Responsibilities	Timing/Context
**Gastroenterologist**	Confirm diagnosis, assess clinical improvement and mucosal healing, manage complications (e.g., refractory celiac disease, malignancy risk), coordinate care	Baseline, 3, 6, and 12 months after diagnosis, then every 6–12 months thereafter or as clinically indicated
**Dietitian**	Provide education on gluten-free diet (GFD), assess nutritional adequacy, monitor for deficiencies, reinforce adherence strategies	At diagnosis, 6–12 months, and ongoing as needed
**Primary Care Provider**	Preventive health (vaccinations, cardiovascular/metabolic risk), screen for comorbidities (osteoporosis, thyroid disease, diabetes), ensure continuity of care	Ongoing, annual wellness
**Psychologist/Psychiatrist/Mental Health Professional**	Screen for anxiety, depression, eating disorders, or maladaptive behaviors (e.g., ARFID, excessive fear of gluten), provide counseling	At diagnosis if risk factors present, or as needed
**Endocrinologist**	Evaluate and manage bone health in patients with osteopenia or osteoporosis, monitor calcium, vitamin D, and parathyroid hormone levels, manage associated endocrine comorbidities (e.g., thyroid disease, diabetes), guide therapy when indicated.	As indicated by abnormal bone density or metabolic findings.
**Pharmacist**	Review medications and supplements for hidden gluten, provide counseling on safe formulations, collaborate with prescribers to ensure gluten-free options	Baseline and when new medications are prescribed.

**Table 2:** Common Challenges with the Gluten-Free Diet in Celiac Disease: Clinical Clues and Suggested Management Strategies.

Challenge	Symptoms/Things to Ask About in Clinic	Suggested Management *
High salt content in gluten-free processed foods	Elevated blood pressure, headaches, patient-reported high salt intake, edema	Check BP regularly Counsel on sodium intake and label literacy Encourage whole foods
High sugar and saturated fat content	Weight gain, elevated HbA1c, dyslipidemia	Screen HbA1c and lipid panel annually Advise to limit ultra-processed foods
Low fiber content	Constipation, incomplete evacuation of bowel movements, low stool frequency, ask about whole grain intake	Promote naturally gluten-free high-fiber foods (e.g., quinoa, lentils) Consider fiber supplements
Low in essential vitamins and minerals	Fatigue, pallor, neuropathy, bone pain, hair loss; ask about supplement use and food variety	Test iron, B12, D, folate, zinc, copper at diagnosis and annually if risk persists; supplement as needed
Increased caloric density leading to weight gain/obesity	Weight trends, BMI increase, discuss satiety and portion sizes, lifestyle activity level, changes in fit of clothing	Track weight and waist circumference at every visit Personalize calorie and meal plans If overweight or obese, monitor for metabolic syndrome associated complications (e.g., metabolic-dysfunction-associated liver disease), consider transient elastography of liver Consider weight management strategies including weight-loss medications, metabolic and bariatric endoscopy and surgery
Psychological distress and food-related anxiety	Fear of contamination, avoidance of social meals, restrictive eating habits, mood symptoms	Screen for disordered eating and eating disorders Refer to behavioral health, psychology or psychiatry if needed Provide balanced, non-alarmist counseling
Higher cost and reduced availability of gluten-free products	Financial stress, skipped meals, reliance on limited food options, ask about food insecurity	Refer to social work Suggest budget-friendly GFD staples Educate on affordable nutrition
Social isolation and stigma	Avoidance of travel/restaurants, reluctance to eat outside home, emotional burden of diagnosis	Validate social/emotional challenges Connect with support groups Suggest coping strategies
Frequent unintentional gluten exposure	GI symptoms despite reported adherence, inconsistent symptom patterns, unclear label reading	Educate on cross-contamination and reading labels Consider GIP stool/urine testing for awareness and adherence

* All recommendations are based on expert consensus, standard clinical practice, and observational evidence rather than randomized controlled trials. The strength of evidence across these areas is generally low to moderate and should be interpreted within the context of individual patient circumstances.

## Data Availability

Data sharing is not applicable.
